# Clinical Lecture—Mercy Hospital

**Published:** 1861-01

**Authors:** N. S. Davis

**Affiliations:** Prof. of Practical Medicine and of Clinical Medicine


### MERCY HOSPITAL.

Service of Prof. N. S. DAVIS, M. D., Prof. of Practical Medicine and of Clinical Medicine.

(Reported by FRANK W. REILLY, Senior Class Med. Depart. Lind University.)

*Traumatic Spinal Curvature—Paraplegia and Anæsthesia.* —GENTLEMEN :—Our time this morning will be occupied principally—perhaps exclusively—with a case of injury to the spine, with loss of muscular action—paralysis and sensation—not total, however, at this time, for the patient is able to control motion to some extent, and, if supported, can even walk across the room. Some degree of anæsthesia yet exists in one, if not both extremeties, though to a very considerably less extent than immediately after the accident.

The history of the case is briefly as follows :

The patient, a young girl about seventeen years of age, was sitting sewing in a room, with her back obliquely to a partition wall, separating another room, in which persons were handling a gun. This being accidentally discharged, the ramrod from the barrel, was driven partially through the wall into the patient's back, impaling her upon the point. The wound thus produced, was situated between the inferior angle of the scapula and the corresponding dorsal vertebræ, in a forward and lateral direction, tearing the latissimus dorsi and trapezius muscles. The rod was found to be broken, on withdrawing it, and the wound carefully searched for splinters, &c., which were supposed to be all removed, but both the patient and her father insist that, subsequently, a fragment found its way into the intestine, and was passed by the rectum. I doubt, however, from the situation of the wound, and the very serious and extensive lesion, this would necessitate, if such were the case. Still it is not impossible that a splinter might have been deflected by a rib, partially penetrated the colon, which, if the stomach was empty, might rise sufficiently high to receive it, there become impacted in such a way as to prevent the formation of an opening between the external wound and the intestine, and after a time have suppurated through, and been discharged. The probabilities, however, are against it.

The accident was followed by paralysis, and the patient remained in a precarious and helpless condition for some time; confined to bed, the usual gangrene of the nates and hips occurred—bed sores, one of which is not yet healed. This occurred something over a year since, and though the wound healed gradually and kindly, and the usual organic functions were in a great degree restored, she has been stationary for some three or four months past. During her confinement to bed, although no trace of injury to the spine can be discovered, such a degree of lateral spinal curvature took place as to alarm herself and friends, and induce her to seek treatment here. The vertebral column does not seem to have been directly injured—the rod evidently having struck outside the bodies of the vertebræ and pursuing the course before stated. The paralysis and loss of sensation in the extremities, is due to the pressure of lymph, effused into the sheath of the spinal cord and nerves, during the inflammation of the adjacent parts. The lateral curvature is best explained by the disturbed equilibrium of the muscular forces; those on the wounded side yielding, and the antagonistic ones contracting with a possibly increased vigor. And here it may be as well to review briefly the subject of spinal curvatures. These are of two kinds; the first and least serious—the true simple curvature in which the curve is the segment of the arc of a circle, either large or small, according to the degree of curvature. The other and graver, not a curve at all, but an angle, either greater or less according to the extent of the column and number of vertebræ involved. These may both occur in various directions, either anteriorly, laterally or posteriorly, though this last is of very rare occurrence. I have never seen a case of spinal curvature with the convexity of the curve looking forwards, (constituting a true posterior curvature,) and you will very readily see how difficult this would be. In angular curvature, as it is termed, the disease consists in the destruction of parts of the vertebræ, and while these are spongy and porous, and separated by cartilage, anteriorly, posteriorly, you will remember, the structure is dense and hard from the formation of processes, &c. Hence, posterior curvatures are rare from the greater ease with which caries, necrosis, ulcerative absorption, &c., progress in spongy porous bones, cartilages, &c. The disease, *e. g.* necrosis or caries, will commence in the body of the bone, or the intervertebral substance will be destroyed by ulcerative absorption, just as the cartilage of the knee joint may be destroyed, bone exposed and attacked, and when the bodies are destroyed wholly or partially, the column shuts down forward, and the angle is produced; as when by the partial withdrawal of the middle finger you allow the index and ring fingers to approximate. (*illustrated with the fingers*). The spinous processes jut out, and the whole appearance is too plain to be mistaken. This destruction of the bodies and consequent tipping of the spinal column forwards, causes the patient to instinctively throw his head backwards in order to keep his balance, and thus we get the S form of spine in angular curvature. It is easily recognized, and you will not be apt to mistake it. Such cases are common enough among scrofulous children; and as one, now and then, grows up, you will occasionally meet on the streets, persons with the head thrown back—fixed, and thrown back, the neck apparently very short, shoulders high, and thrown forwards, sometimes up to the ears almost, an angular protuberance between the scapulæ, and the body disproportionately short, compared with the extremities, and this last most noticable, usually, in the superior extremities, so that they seem to be unusually long-armed. I remember three such cases of ladies in my own practice.

The disease very rarely occurs in adults; mostly in children, in whom it commences usually by slow inflammation, ending in ulcerative absorption of the intervertebral substance, after which the bodies are attacked, and are gradually absorbed. The adjoining bodies now fall together; that organ in which resides the wonderful power, the *reflex function*, by which the automatic movements are regulated, and by which the medulla oblongata and other portions of the brain, whose office it is to generate and co-ordinate the agency of voluntary motion, are enabled to transmit their power to the most remote extremity, this telegraph of the human system, the spinal cord, is compressed, the medium of communication is cut off, and all that part of the system supplied with nerve fibre below the point of compression is bereft of sensation and of voluntary motion—not suddenly, without the compression is sudden and marked; but at first the lower extremities get clumsy, the child trips and stumbles easily, is restless at night, cries out often, and without apparent cause; then the mother or nurse notices that the child stoops, complains when taken hold of; gets alarmed and sends for the physician, who finds, usually, the characteristic angle, with its row of processes sharp and prominent as the knuckles of a clenched fist.

If this is taken in hand at once, much may be done to remedy the complaint. The horizontal position should be strongly insisted upon,—let the child roll and toss and tumble as much as it chooses, but keep it horizontal. A gentle uniform counter-irritant will be found beneficial; usually a pitch plaster, (*Pix abietis*, Ph. U. S.,) with points of tartarized antimony upon it, will suffice. The diet and internal treatment will also demand your attention. The first should be gentle and nutritious, and the second the usual treatment of scrofulous inflammations. All excitement, stimulants, &c., should be avoided; and usually, if the treatment is commenced sufficiently early and faithfully followed, the disease may be arrested in from six to eight weeks, during the latter part of which the confinement to the horizontal position may be relaxed, and the patient allowed to sit up an hour or two each day. The employment of an apparatus to relieve the affected portion of the spine of the weight of the head and shoulders, by transferring it through the instrument, to the pelvis, will now be found advisable; and by the use of this and a due regard to habitual position and subsequent health, the disease may not only be arrested and cured, but in many cases the deformity will not be perceptible. In most cases, however, the disease is allowed to make too much progress before it is interfered with; and in such, the destruction of the bodies, frequently to such an extent as to cause compression of the cord, and the consequent change of position cannot be remedied; though by the arrest of inflammation, use of instrument, &c., the sufferer may be restored to a comparative degree of health.

Another variety of angular curvature, and one much more usually fatal and distressing than the one we have just been discussing, arises, not from simple scrofulous disease, but is the result of tuberculous deposit in the bodies of the vertebræ; following which we have the slow development of caries, the bodies growing soft, the inflammation aggravated by pressure, absorption taking place, and in from three to six months, usually, after patient begins to complain, there will be formation of an abscess, which will depend upon the situation of the diseased bone, for its external manifestation. Occurring upon the anterior aspect of the bodies, if high up its discharge externally, will be slow, finding its way out either through the intercostal spaces, or the cruræ of the diaphragm and down along the psoas muscle, where it may appear in the lumbar region, or under Poupart's ligament in the groin. If the lower dorsal and lumbar vertebræ are involved, the abscess may point over the crest of the ilium, or under Poupart's ligament in the upper part of the thigh.

I have said that this variety of spinal disease is usually fatal, and you will find it as uniformly so as is pulmonary tuberculosis. It will usually run its course in five or six years, though as in other varieties of tuberculosis, the patient's life may be protracted by judicious treatment beyond this. But it will surely, no matter how gradually, wear the patient out by the constant drain upon the system. Occasionally the abscess and its channel will contract, the discharge cease, and the patient and friends will flatter themselves that a cure is being effected; but sooner or later the discharge will be renewed, and abscess distend, and this may occur again and again, until in the advanced stages, hectic fever, night sweats and colliquative diarrhœa supervene and carry off the patient. I should have mentioned that your diagnosis may be materially assisted by an examination of the other organs of the body, when you will usually find tuberculous deposit to exist in the lungs.

Simple spinal curvature rarely depends on disease of the vertebræ; but is almost invariably the result of deficient muscular action. The position, construction and offices of the spinal column are such as to demand a strong well-balanced muscular power, constantly at work in order to maintain it in its symmetrical integrity and usefulness. The motions of the head, shoulders and arms, constantly changing, and in that change affecting the spine, upon which their weight is superimposed, would speedily destroy these conditions, should this muscular power be, from any cause, illy balanced. And one of the most common causes of this disturbed equilibrium is to be found in the compression of dresses; the waist of the dress comes over the bellies of the associated erector spinæ muscle, at a point where it should have the greatest amount of contractile power; this pressure impairs action, the muscles lose their force, grow weak, allow the spine to yield to the forward motion of the head, and the child—for it is noticeable usually only in children—begins to get round or stoop-shouldered—hunch-backed; and all the antagonistic muscles of the abdomen, the psoas and the quadratus, will serve still further to increase this.

Another very common cause of curvature is to be found in an unequal exercise of the superior extremities; and in habitual faulty positions, familiar examples of which may be found in almost every school girl or boy; the resting of the head on one arm on the desk, day after day, bending over books, with the muscles of one side unduly extended, and of the other relaxed; standing with the weight of the body thrown on one leg, thus elevating the pelvis on the corresponding, and the shoulder of the opposite side; the wearing of low-necked dresses or of underclothing with shoulder-straps, by which the child is tempted to let the strap or dress fall from one shoulder and hitch it up on the other, is also a fruitful source of this evil. In any or all of these ways, you may have the commencement of lateral curvature, the indications of which are the dropping of one, and the undue prominence of the other shoulder, and the projection of one scapula or of one side of the breast; and on examining the child you will find the spine curved laterally and the shoulder and hip of opposite sides more or less distorted. This loss of the true vertical direction is now increased by the weight of the head and shoulders, and also by the action of the muscles, the dorsal on the concave side acting like the cord of a bow to draw the ends together, while the trapezius, levator anguli scapulæ, and rhomboidii exert direct traction on the periphery of the curve above, and the latissimus dorsi below. And if this is not checked, the deformity will increase as the child grows up, until it will require a considerable amount of cotton and padding to get up a symmetrical bust, at the time when this is a desideratum to girls.

I will not stop here to dwell on the treatment of this latter class of cases. Vigilant care in guarding against faulty positions, judicious exercise, fresh air and attention to the general health are points so obvious as to suggest themselves at once to your minds.

Mechanical injury, has in the case we are about to examine, done suddenly what we have seen is usually effected in the slow course of events. The extent of the cicatrix will show that the wound must have very severely mangled the muscles on the convex side of the curve, while those on the concave remaining sound and vigorous, the commencement of the curve took place, and was augmented, as soon as she became able to sit up, by the weight of the head and upper extremities. The complication of paralysis is the result of positive inflammation of the spinal cord and nerves of the injured side, without fracture of the spine, or compression of the cord, other than that produced by the effusion, plastic or serous, attendant on the inflammation. It is probable that this effusion has become, at least, partially absorbed, inasmuch as the degree of paralysis is decidedly less now than immediately after the inflammation. The action of the nervous system is here beautifully illustrated—pressure upon the seat of the injury producing pain, which is referred to the extremities, which these nerves supply.

The indications are to allay the morbid sensitiveness to pressure at the point of injury by narcotic applications, and by the use of a proper instrument, to relieve the spine of the weight of the head and shoulders. Also to devise means by which she may exercise her lower extremities, with a view to overcoming the partial paralysis now existing. For these purposes we shall direct a plaster of belladonna to the dorsal spine; an instrument, consisting of a cushioned band around the hips, a rod on each side terminated with a crutch under the arms, a flat steel strap up each side of the spine, and shoulder straps, so that when well fitted it aids in holding the spine erect, and transfers much of the weight of the head and shoulders, directly to the pelvis, without baring on the curved part of the spine; and then direct the assistants to aid her in regular, systematic attempts to walk, two or three times a day. Experience has demonstrated that much may be gained in the treatment of paralyzed parts by regular and persevering efforts to exercise the affected muscles.

As this patient is also considerably anæmic and debilitated, we shall give her internally, three times a day, a teaspoonful of the following, viz:

| | | |
|---|---|---|
| ℞ | Strychnine, | 1 gr. |
| | Citrate of Iron, | ʒ j. |
| | Citric Acid, | ʒ j. |
| | Water, | ℥ ij. |

